# Occupational inequalities in mortality in Korea: an analysis using nationally representative mortality follow-up data from the late 2000s and after

**DOI:** 10.4178/epih.e2022038

**Published:** 2022-04-06

**Authors:** Eunjeong Noh, Young-Ho Khang

**Affiliations:** 1 Institute of Health Policy and Management, Seoul National University Medical Research Center, Seoul, Korea; 2 Department of Health Policy and Management, Seoul National University College of Medicine, Seoul, Korea

**Keywords:** Health inequalities, Mortality, Occupations, Republic of Korea, Socioeconomic factors

## Abstract

Many Korean and international studies have found higher mortality rates and poorer health conditions among manual workers than among non-manual workers. However, a recent study using unlinked data argued that since the economic crisis in Korea in the late 2000s, the mortality estimates of male Korean non-manual workers have been higher than those of manual workers. Our work using individually linked data from the late 2000s and after aimed to examine mortality inequality by occupational class. We analyzed Korea National Health and Nutrition Examination Survey data that were individually linked to cause-of-death data. Cox regression analysis was used to identify the hazard ratios for mortality by occupational class. Of 11,766 males aged between 35 and 64, 397 died between 2007 and 2018: 142 died from cancer, 68 from cardiovascular disease, 88 from external causes, and 99 from other causes. After controlling for age, the mortality estimates for manual workers were 1.85 times higher than those for upper non-manual workers (p<0.05). We observed no evidence of reversed mortality inequality among occupational classes in Korea since the 2000s; this previously reported finding might have been due to numerator-denominator bias arising from the use of unlinked data.

## INTRODUCTION

Non-manual workers are known to have better health and lower mortality rates than manual workers [1-7]. However, a recent study using unlinked data reported that this general pattern appeared to be different in Korea and Japan [8]. That study claimed that non-manual workers’ mortality estimates among Korean males aged 35-64 have risen since the 2000s economic crisis, becoming higher than those of manual workers. This purported reversed occupational inequality in mortality could be attributed to a sharp increase in cancer-related and suicide-related deaths among non-manual workers since the economic crisis [8]. The authors of the study suggested that the national economic crisis in the late 2000s might have negatively influenced the health risk factors affecting non-manual workers’ mortality. However, another recent study using data on males aged 35-64—the same sex and age group used in the prior study [8]—in Korea from 2007 to 2009 found that socioeconomic indicators, such as education, income, parental education, and economic activity, as well as work environment indicators, were more favorable among non-manual workers than among manual workers, and the prevalence of unfavorable health-related behavioral indicators, such as smoking, high-risk alcohol consumption, depression, and suicidal ideation, was still higher among manual workers than among their counterparts [9]. The previously reported finding of a reversed pattern of mortality inequality among occupational classes in Korea may have been caused by numerator-denominator bias, since the study used population and death counts from different unlinked data sources [9-12]. Although individually linked data from the late 1990s and early 2000s in Korea have provided evidence for occupational mortality inequalities favoring non-manual workers [5,13,14], no research has been done using individually linked cohort data from the late 2000s. An analysis of linked cohort data from the late 2000s to the late 2010s could help determine whether there is, in fact, a reversed pattern of occupational inequality in mortality in Korea. This study aimed to determine mortality estimates by occupational class in Korea since the 2000s.

## MATERIALS AND METHODS

### Population and sample

This study linked data from the 2007-2015 Korea National Health and Nutrition Examination Surveys (KNHANES) to the 2007-2018 cause-of-death data provided by the Korea Disease Control and Prevention Agency (KDCA). The KNHANES is an annual nationwide survey conducted to understand the health and nutritional status of Korean citizens based on Section 16 of the National Health Promotion Act [15]. Of the 53,101 KNHANES respondents from 2007 to 2015, 51,603 (22,083 males, 29,520 females) consented to linkage with cause-of-death data and had valid unique personal identification numbers. Among them, 12,505 males aged 35-64 were eligible for the study purpose, as a prior study examined occupational mortality inequality among males aged 35-64 [8]. With the exclusion of 739 individuals due to missing information on main indicators, the final sample included 11,766 individuals [15]. Individual data linkage was performed internally at the KDCA, and data without any personal information were provided for our analysis.

### Variables

The occupational information provided by the KNHANES from 2007 to 2015 was the independent variable. Four occupational groups were identified based on the same definitions used by Tanaka et al. [8]: the upper non-manual group included managers and professionals; the lower non-manual group included clerks and service and sales workers; the manual group included craft workers, plant and machine operators and assemblers, and elementary occupations; and the “others” group included workers in other jobs (e.g., agricultural, forestry and fishery workers and unemployed individuals). Data on all-cause and cause-specific deaths by occupational class between 2007 and 2018 were identified. Four major causes of death were considered, including cancer (International Classification of Diseases, 10th revision [ICD-10] codes: C00-D48), cardiovascular disease (ICD-10 codes: I00-I99), external causes (ICD-10 codes: V01-Y89), and other causes.

### Statistical analysis

Using information on survival time and death status, Cox proportional hazards regression models were employed to analyze the hazard ratios (HRs) of all-cause and cause-specific mortality among occupational classes, after adjusting for age.

### Ethics statement

The study protocol was approved by the Institutional Review Board (IRB) of Seoul National University Hospital (IRB No. E-2006-058-1131). Informed consent was waived by the IRB.

## RESULTS

From 2007 to 2018, 397 of the 11,766 individuals died (3.4%): 142 (35.8%) died from cancer (ICD-10 codes: C00-D48), 68 (17.1%) from cardiovascular diseases (I00-I99), 88 (22.2%) from external causes (V01-Y89), and 99 (24.9%) from other causes. The mean±standard deviation (SD) age of all subjects was 47.84±10.48 years, and the mean follow-up period for all subjects was 7.40±4.89 years. Table 1 presents the mean age and mean follow-up period by occupational class. The mean age was similar among upper non-manual and lower non-manual workers, but relatively older among manual workers and the “others” group. The mean follow-up period by occupational class showed a graded pattern reflecting mortality risk differentials among occupational classes. The all-cause mortality estimate of the manual workers after age adjustment was 1.85 times higher than those of upper non-manual workers (p< 0.05). The HR of lower non-manual workers tended to be higher than that of upper non-manual workers (Table 1).

Age stratified analyses (Supplementary Material 1) showed that the mortality risk of manual workers was generally greater than that of non-manual workers among both males aged 35-49 and males aged 50-64. In Korean females aged 35-64, the mortality differentials between manual and non-manual workers were not significant, partly because of the small numbers of deaths, but the mortality risk of manual workers tended to be greater than that of non-manual workers (Supplementary Material 2).

The cause-specific analysis results showed that, largely because of the small numbers of cause-specific deaths, manual workers did not have statistically significantly higher cause-specific mortality estimates than upper non-manual workers. However, those in the “others” group, who were not classified as manual workers but were either agricultural, forestry, or fishery workers or were unemployed, had much higher mortality estimates (all statistically significant) than upper non-manual workers (Table 2).

## DISCUSSION

This study utilized cohort data that linked the 2007-2015 KNHANES data with 2007-2018 cause-of-death data. By tracking deaths and the causes of death in Korean males aged 35-64, all-cause mortality estimates according to occupational class and mortality estimates based on 4 causes of death were derived. Even after the economic crisis in the late 2000s, the mortality of male Korean manual workers aged 35-64 remained higher than that of non-manual workers. Therefore, there was no evidence to support the finding of reversed mortality inequality by occupational class reported by the previous study [8], which used unlinked census and death registry data. Our study is consistent with the universal conclusions of previous domestic and international studies using mortality follow-up data, which have shown that the mortality estimates of manual workers are higher than those of nonmanual workers [1-7].

Additionally, the prior study proposed that the Korean economic crisis in the 2000s could have negatively affected non-manual workers’ psychosocial health factors, such as stress and depression, which might have contributed to increased mortality due to suicide and cancer [8]. However, although cause-specific mortality estimates were not statistically significantly higher in manual workers than in non-manual workers due to the small numbers of cause-specific deaths in the present study, the HRs for cancer and external causes tended to be higher in manual workers than in non-manual workers (Table 2). The mortality estimates of the “others” group were exceptionally higher than those of non-manual workers, and this discrepancy was statistically significant. Moreover, our recent study using data from the late 2000s showed that psychosocial factors (e.g., depression, stress, suicidal ideation), work environment, and health behaviors related to cancer (e.g., smoking, high-risk alcohol consumption) were more favorable in non-manual workers than in manual workers [9].

In summary, like numerous previous studies on mortality and the prevalence of health risk factors by occupational class, this study found that in Korea, manual workers continued to have higher mortality rates than non-manual workers in the late 2000s and after. This result contradicts the prior claim of reversed mortality inequality by occupational class in Korea, which might be attributed to the denominator-numerator bias that has been commonly raised as a concern in health inequality research using unlinked data. If the census and death data by occupational class are not individually linked, as was the case in the study that proposed the inequality reversal, it is highly likely that the mortality estimates of non-manual workers will be overestimated and those of manual workers underestimated [9-12]. Analyzing the linked data provided via the nationally representative KNHANES (2007-2015) and individual mortality data (2007-2018), this study confirmed that even after the economic crisis of the 2000s, patterns of mortality inequality in Korea remain disadvantageous for those in lower occupational classes.

## Figures and Tables

**Table 1.** Numbers of subjects and deaths, mean age, mean follow-up duration, and age-adjusted HRs and their 95% CIs for all-cause mortality^1^ by occupational class in Korean males aged 35 to 64 (n=11,766)

Variables	No. of subjects	No. of deaths	Age, mean±SD (yr)	Follow-up duration, mean±SD (yr)	HR (95% CI)	p-value
Non-manual						
Upper	2,276	35	45.68±8.49	7.50±4.03	1.00 (reference)	
Lower	3,069	52	45.34±8.68	7.40±3.87	1.31 (0.81, 2.13)	0.28
Manual	3,931	121	48.28±9.34	7.37±4.23	1.85 (1.20, 2.85)	0.01
Others	2,490	189	52.97±9.97	7.35±4.36	3.38 (2.14, 5.33)	<0.001

HR, hazard ratio; CI, confidence interval; SD, standard deviation.

1 Mortality follow-up data from the 2007 and 2015 Korea National Health and Nutrition Examination Surveys.

**Table 2.** Age-adjusted HRs and their 95% CIs for mortality^1^ from 4 broad causes by occupational class in Korean males aged 35 to 64 (n=11,766)

Variables	Non-manual		Manual	Others
	Upper	Lower		
No. of subjects	2,276	3,069	3,931	2,490
No. of cancer deaths	14	17	34	77
HR (95% CI)	1.00 (reference)	1.09 (0.47, 2.50)	1.34 (0.62, 2.90)	2.39 (1.19, 4.79)
p-value		0.85	0.46	0.01
No. of cardiovascular deaths	5	10	21	32
HR (95% CI)	1.00 (reference)	1.46 (0.47, 4.53)	2.06 (0.74, 5.74)	3.25 (1.10, 9.57)
p-value		0.52	0.17	0.03
No. of external causes	10	18	34	26
HR (95% CI)	1.00 (reference)	1.56 (0.67, 3.60)	2.07 (0.95, 4.51)	3.17 (1.25, 8.06)
p-value		0.30	0.07	0.02
No. of other deaths	6	7	32	54
HR (95% CI)	1.00 (reference)	1.11 (0.30, 4.16)	2.53 (0.80, 7.98)	5.79 (1.80, 18.64)
p-value		0.87	0.11	<0.001

HR, hazard ratio; CI, confidence interval.

1 Mortality follow-up data from the 2007 and 2015 Korea National Health and Nutrition Examination Surveys.

## SUPPLEMENTARY MATERIALS

Supplementary Material 1. Age-adjusted hazard ratios (HRs) and their 95% confidence intervals (CIs) of all-cause mortality by occupational class among Korean males aged 35-49 and 50-64 (N = 11,766): mortality follow-up data from the 2007 and 2015 Korea National Health and Nutrition Examination Surveys

Supplementary Material 2. Age-adjusted hazard ratios (HRs) and their 95% confidence intervals (CIs) of all-cause mortality by occupational class in Korean females aged 35-64 (N = 15,798): mortality follow-up data from the 2007 and 2015 Korea National Health and Nutrition Examination Surveys
