# Supplementary material for: Occupational inequalities in mortality in Korea: an analysis using nationally representative mortality follow-up data from the late 2000s and after
Source: Epidemiol Health. 2022 Apr 6;44:e2022038. doi: 10.4178/epih.e2022038 (PMC9350417; doi:10.4178/epih.e2022038)
Supplement: Supplementary Material 1. — Age-adjusted hazard ratios (HRs) and their 95% confidence intervals (CIs) of all-cause mortality by occupational class among Korean males aged 35-49 and 50-64 (N = 11,766): mortality follow-up data from the 2007 and 2015 Korea National Health and Nutrition Examination Surveys [file epih-44-e2022038-suppl1.docx]

Supplementary Material 1. Age-adjusted hazard ratios (HRs) and their 95% confidence intervals (CIs) of all-cause mortality by occupational class among Korean males aged 35-49 and 50-64 (N = 11,766): mortality follow-up data from the 2007 and 2015 Korea National Health and Nutrition Examination Surveys

| Age  groups | Occupational Class | No. of  Subject | No. of  Death | HR  (95% CI) | *P*-value |
| --- | --- | --- | --- | --- | --- |
| 35-49 | Upper non-manual | 1493 | 13 | 1.00 | Reference |
|  | Lower non-manual | 2078 | 21 | 1.26  (0.59-2.67) | 0.55 |
|  | Manual | 1932 | 38 | 2.64  (1.30-5.35) | 0.01 |
|  | Others | 582 | 26 | 5.75  (2.74-12.06) | <.001 |
| 50-64 | Upper non-manual | 783 | 22 | 1.00 | Reference |
|  | Lower non-manual | 991 | 31 | 1.35  (0.73-2.53) | 0.34 |
|  | Manual | 1999 | 83 | 1.52  (0.91-2.52) | 0.11 |
|  | Others | 1908 | 163 | 3.04  (1.83-5.04) | <.001 |
