# Supplementary material for: Occupational inequalities in mortality in Korea: an analysis using nationally representative mortality follow-up data from the late 2000s and after
Source: Epidemiol Health. 2022 Apr 6;44:e2022038. doi: 10.4178/epih.e2022038 (PMC9350417; doi:10.4178/epih.e2022038)
Supplement: Supplementary Material 2. — Age-adjusted hazard ratios (HRs) and their 95% confidence intervals (CIs) of all-cause mortality by occupational class in Korean females aged 35-64 (N = 15,798): mortality follow-up data from the 2007 and 2015 Korea National Health and Nutrition Examination Surveys [file epih-44-e2022038-suppl2.docx]

Supplementary Material 2. Age-adjusted hazard ratios (HRs) and their 95% confidence intervals (CIs) of all-cause mortality by occupational class in Korean females aged 35-64 (N = 15,798): mortality follow-up data from the 2007 and 2015 Korea National Health and Nutrition Examination Surveys

|  | No. of subjects | No. of deaths | HR  (95% CI) | *P*-value |
| --- | --- | --- | --- | --- |
| Upper non-manual | 1541 | 9 | 1.00 | Reference |
| Lower non-manual | 3793 | 25 | 0.84  (0.34-2.08) | 0.70 |
| Manual | 2357 | 37 | 1.26  (0.53-2.98) | 0.60 |
| Others | 8107 | 145 | 1.59  (0.72-3.54) | 0.26 |
